# 1-Methyl-2-[(*E*)-2-(2-thien­yl)ethen­yl]quinolinium 4-bromo­benzene­sulfonate

**DOI:** 10.1107/S1600536810007488

**Published:** 2010-04-10

**Authors:** Hoong-Kun Fun, Thawanrat Kobkeatthawin, Suchada Chantrapromma

**Affiliations:** a X-ray Crystallography Unit, School of Physics, Universiti Sains Malaysia, 11800 USM, Penang, Malaysia; b Crystal Materials Research Unit, Department of Chemistry, Faculty of Science, Prince of Songkla University, Hat-Yai, Songkhla 90112, Thailand

## Abstract

In the title compound, C_16_H_14_NS^+^·C_6_H_4_BrO_3_S^−^, the cation exists in an *E* configuration and is essentially planar, the dihedral angle between the quinolinium and thio­phene rings being 3.45 (9)°. The anion is inclined to the cation with dihedral angles of 75.43 (8) and 72.03 (11)°, respectively between the benzene ring and the quinolinium and thio­phene rings. In the crystal, the cations and anions are arranged individually into separate chains along the *c* axis. The cation chains are stacked in an anti­parallel manner along the *a* axis by π⋯π inter­actions with centroid–centroid distances of 3.7257 (13) and 3.7262 (14) Å. Weak C—H⋯O and C—H⋯π inter­actions link the cations and anions into a three-dimensional network. Short Br⋯S [3.7224 (5) Å] and Br⋯O [3.4267 (16) Å] contacts are also observed.

## Related literature

For bond-length data, see: Allen *et al.* (1987 ▶). For background to non-linear optical materials research, see: Chantrapromma *et al.* (2009*a* ▶,*b* ▶), Fun *et al.* (2009 ▶); Raimundo *et al.* (2002 ▶). For related structures, see: Chantrapromma *et al.* (2006 ▶); Ruanwas *et al.* (2008 ▶). For the stability of the temperature controller used in the data collection, see Cosier & Glazer, (1986 ▶).
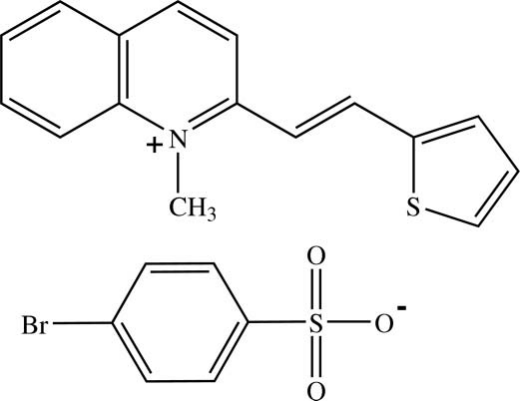


## Experimental

### 

#### Crystal data

C_16_H_14_NS^+^·C_6_H_4_BrO_3_S^−^
*M* *_r_* = 488.41
Monoclinic, 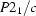

*a* = 7.9026 (1) Å
*b* = 18.8211 (2) Å
*c* = 13.4816 (1) Å
β = 93.292 (1)°
*V* = 2001.89 (4) Å^3^
*Z* = 4
Mo *K*α radiation
μ = 2.29 mm^−1^
*T* = 100 K
0.34 × 0.32 × 0.24 mm

#### Data collection

Bruker APEXII CCD area detector diffractometer
Absorption correction: multi-scan (*SADABS*; Bruker, 2005 ▶) *T*_min_ = 0.511, *T*_max_ = 0.612
51446 measured reflections
5827 independent reflections
5163 reflections with *I* > 2σ(*I*)
*R*_int_ = 0.030

#### Refinement

*R*[*F* ^2^ > 2σ(*F* ^2^)] = 0.035
*wR*(*F* ^2^) = 0.089
*S* = 1.12
5827 reflections
263 parameters
H-atom parameters constrained
Δρ_max_ = 1.18 e Å^−3^
Δρ_min_ = −0.46 e Å^−3^

### 

Data collection: *APEX2* (Bruker, 2005 ▶); cell refinement: *SAINT* (Bruker, 2005 ▶); data reduction: *SAINT*; program(s) used to solve structure: *SHELXTL* (Sheldrick, 2008 ▶); program(s) used to refine structure: *SHELXTL*; molecular graphics: *SHELXTL*; software used to prepare material for publication: *SHELXTL* and *PLATON* (Spek, 2009 ▶).

## Supplementary Material

Crystal structure: contains datablocks global, I. DOI: 10.1107/S1600536810007488/sj2736sup1.cif

Structure factors: contains datablocks I. DOI: 10.1107/S1600536810007488/sj2736Isup2.hkl

Additional supplementary materials:  crystallographic information; 3D view; checkCIF report

## Figures and Tables

**Table 1:** Hydrogen-bond geometry (Å, °) *Cg*4 is the centroid of the C17–C22 benzene ring.

*D*—H⋯*A*	*D*—H	H⋯*A*	*D*⋯*A*	*D*—H⋯*A*
C3—H3*A*⋯O3^i^	0.93	2.51	3.409 (3)	162
C7—H7*A*⋯O1^ii^	0.93	2.47	3.241 (3)	140
C8—H8*A*⋯O2^iii^	0.93	2.29	3.218 (3)	175
C10—H10*A*⋯S1	0.93	2.77	3.185 (2)	108
C11—H11*A*⋯O2^iii^	0.93	2.31	3.238 (3)	176
C15—H15*A*⋯O3^iv^	0.93	2.40	3.265 (3)	154
C16—H16*B*⋯O1^v^	0.96	2.43	3.321 (3)	155
C17—H17*A*⋯O1	0.93	2.54	2.915 (3)	105
C20—H20*A*⋯O1^vi^	0.93	2.32	3.242 (3)	172
C13—H13*A*⋯*Cg*4^iii^	0.93	2.57	3.434 (2)	155

Symmetry codes: (i) 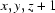; (ii) 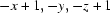; (iii) 
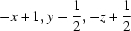
; (iv) 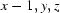; (v) 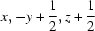; (vi) 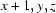.

**Fig. 1.**
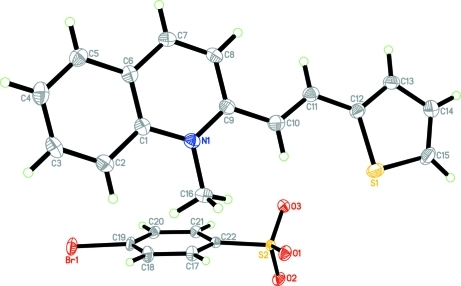
The molecular structure of the title compound, with 50% probability displacement ellipsoids and the atom-numbering scheme.

**Fig. 2.**
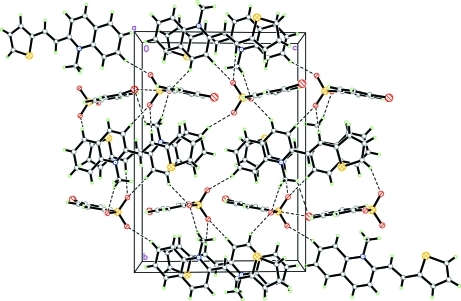
The crystal packing of the title compound viewed down the a axis. Hydrogen bonds and weak C—H···O interactions are shown as dashed lines.

## supplementary crystallographic information

### Comment

During the course of our NLO (non-linear optical) materials research, we have previously reported the crystal structures of the NLO-active compounds (Chantrapromma *et al.*, 2009*a*, *b*; Fun *et al*., 2009). With the knowledge that the organic dipolar compounds with extended π systems and having terminal donor and acceptor groups are likely to exhibit large hyperpolarizability (*β*) (Raimundo *et al.*, 2002), the title compound (I) was designed and synthesized in order to study its NLO properties. Unfortunately (I) crystallizes out in a centrosymmetric *P*2_1_/c space group which precluded the second-order nonlinear optical properties.

The asymmetric unit of the title compound (Fig. 1) consists of the C_16_H_14_NS^+^ cation and C_6_H_4_BrO_3_S^-^ anion. The cation exists in the *E* configuration with respect to the C10═C11 double bond [1.348 (3) Å] and is essentially planar with the dihedral angle between the quinolinium and the thiophene rings being 3.45 (9)° and the torsion angles C9–C10–C11–C12 = -179.8 (2)°. The ten non-H atoms of quinolinium unit lie on the same plane with an r.m.s. deviation of 0.0184 (2) Å. The relative arrangement of cation and anion is shown by the angles between the mean plane of the 4-bromophenyl ring and those of the quinolinium and thiophene rings which are 75.43 (8)° and 72.03 (11)°, respectively. The bond lengths are normal (Allen *et al.*, 1987) and are comparable with those in related structures (Chantrapromma *et al.*, 2006; Ruanwas *et al.*, 2008).

In the crystal, all O atoms of sulfonate group are involved in weak C—H···O interactions (Table 1). The cations and anions are arranged individually into chains along the *c* axis (Fig. 2) . The cation chains are stacked in an antiparallel manner along the *a* axis by π–π interactions with Cg_1_···Cg_2_ = 3.7257 (13) Å (symmetry code: -x,-y, 1-z) and  Cg_1_···Cg_3_ = 3.7262 (14) Å (symmetry code: 1-x, -y, 1-z); Cg_1_, Cg_2_ and Cg_3_ are the centroids of the S1/C12–C15, N1/C1/C6–C9 and C1–C6 rings, respectively. Weak C—H···O and C—H···π interactions (Table 1) link the cations and anions into a three-dimensional network; Cg_4_ is the centroid of the C17–C22 benzene ring. Short Br···S [3.7224 (5) Å] and Br···O [3.4267 (16) Å] contacts (symmetry code for both: 1+x, 1/2-y, 1/2+z) are also observed.

### Experimental

2-(2-Thiophenestyryl)-1-methylquinolinium iodide (compound A) was synthesized by mixing a solution (1:1:1 molar ratio) of 1,2-dimethylquinolinium iodide (2.00 g, 7.0 mmol), 2-thiophenecarboxaldehyde (0.64 ml, 7.0 mmol) and piperidine (0.69 ml, 7.0 mmol) in hot methanol (40 ml). The resulting solution was refluxed for 5 h under nitrogen atmosphere. The resultant solid was filtered off and washed with diethyl ether. Silver(I)4-bromobenzenesulfonate (compound B) was synthesized according to our previously reported procedure. (Chantrapromma *et al.*, 2006). The title compound was synthesized by mixing compound A (0.10 g, 0.26 mmol) in hot methanol (50 ml) and compound B (0.09 g, 0.26 mmol) in hot methanol (20 ml). The mixture immediately yielded a grey precipitate of silver iodide. After stirring the mixture for ca. 45 min, the precipitate was removed and the resulting solution was evaporated yielding a brown solid. Brown block-shaped single crystals of the title compound suitable for *x*-ray diffraction analysis were recrystallized from methanol solvent by slow evaporation at room temperature over a few weeks. (Mp. 538–539 K).

### Refinement

All H atoms were positioned geometrically and allowed to ride on their parent atoms, with d(C-H) = 0.93 Å for aromatic and CH and 0.96 Å for CH_3_ atoms. The *U*_iso_ values were constrained to be 1.5*U*_eq_ of the carrier atom for methyl H atoms and 1.2*U*_eq_ for the remaining H atoms. A rotating group model was used for the methyl groups. The highest residual electron density peak is located at 0.75 Å from Br1 and the deepest hole is located at 0.55 Å from S1.

### Crystal data

**Table:**

C_16_H_14_NS^+^·C_6_H_4_BrO_3_S^−^	*F*(000) = 992
*M**_r_* = 488.41	*D*_x_ = 1.620 Mg m^−^^3^
Monoclinic, *P*2_1_/*c*	Melting point = 538–539 K
Hall symbol: -P 2ybc	Mo *K*α radiation, λ = 0.71073 Å
*a* = 7.9026 (1) Å	Cell parameters from 5827 reflections
*b* = 18.8211 (2) Å	θ = 2.2–30.0°
*c* = 13.4816 (1) Å	µ = 2.29 mm^−^^1^
β = 93.292 (1)°	*T* = 100 K
*V* = 2001.89 (4) Å^3^	Block, brown
*Z* = 4	0.34 × 0.32 × 0.24 mm

### Data collection

**Table:**

Bruker APEXII CCD area detector diffractometer	5827 independent reflections
Radiation source: sealed tube	5163 reflections with *I* > 2σ(*I*)
graphite	*R*_int_ = 0.030
φ and ω scans	θ_max_ = 30.0°, θ_min_ = 2.2°
Absorption correction: multi-scan (*SADABS*; Bruker, 2005)	*h* = −11→11
*T*_min_ = 0.511, *T*_max_ = 0.612	*k* = −26→26
51446 measured reflections	*l* = −18→18

### Refinement

**Table:**

Refinement on *F*^2^	Primary atom site location: structure-invariant direct methods
Least-squares matrix: full	Secondary atom site location: difference Fourier map
*R*[*F*^2^ > 2σ(*F*^2^)] = 0.035	Hydrogen site location: inferred from neighbouring sites
*wR*(*F*^2^) = 0.089	H-atom parameters constrained
*S* = 1.12	*w* = 1/[σ^2^(*F*_o_^2^) + (0.0325*P*)^2^ + 3.2344*P*] where *P* = (*F*_o_^2^ + 2*F*_c_^2^)/3
5827 reflections	(Δ/σ)_max_ = 0.001
263 parameters	Δρ_max_ = 1.18 e Å^−^^3^
0 restraints	Δρ_min_ = −0.46 e Å^−^^3^

### Special details

**Table:**

Experimental. The crystal was placed in the cold stream of an Oxford Cryosystems Cobra open-flow nitrogen cryostat (Cosier & Glazer, 1986) operating at 100.0 (1) K.
Geometry. All esds (except the esd in the dihedral angle between two l.s. planes) are estimated using the full covariance matrix. The cell esds are taken into account individually in the estimation of esds in distances, angles and torsion angles; correlations between esds in cell parameters are only used when they are defined by crystal symmetry. An approximate (isotropic) treatment of cell esds is used for estimating esds involving l.s. planes.
Refinement. Refinement of F^2^ against ALL reflections. The weighted R-factor wR and goodness of fit S are based on F^2^, conventional R-factors R are based on F, with F set to zero for negative F^2^. The threshold expression of F^2^ > 2sigma(F^2^) is used only for calculating R-factors(gt) etc. and is not relevant to the choice of reflections for refinement. R-factors based on F^2^ are statistically about twice as large as those based on F, and R- factors based on ALL data will be even larger.

### Fractional atomic coordinates and isotropic or equivalent isotropic displacement parameters (Å^2^)

**Table:**

	*x*	*y*	*z*	*U*_iso_*/*U*_eq_	
S1	0.00270 (7)	0.06224 (3)	0.28565 (4)	0.02261 (11)	
N1	0.3667 (2)	0.04462 (9)	0.63104 (13)	0.0179 (3)	
C1	0.4567 (3)	0.03115 (11)	0.72171 (15)	0.0178 (4)	
C2	0.4926 (3)	0.08556 (12)	0.79170 (16)	0.0219 (4)	
H2A	0.4584	0.1320	0.7780	0.026*	
C3	0.5783 (3)	0.06935 (13)	0.87999 (17)	0.0246 (4)	
H3A	0.6020	0.1053	0.9260	0.030*	
C4	0.6314 (3)	−0.00053 (14)	0.90275 (17)	0.0257 (5)	
H4A	0.6896	−0.0104	0.9631	0.031*	
C5	0.5967 (3)	−0.05415 (12)	0.83528 (16)	0.0220 (4)	
H5A	0.6308	−0.1004	0.8501	0.026*	
C6	0.5094 (3)	−0.03885 (11)	0.74348 (15)	0.0185 (4)	
C7	0.4708 (3)	−0.09311 (11)	0.67292 (16)	0.0198 (4)	
H7A	0.5081	−0.1393	0.6856	0.024*	
C8	0.3799 (3)	−0.07819 (11)	0.58700 (16)	0.0204 (4)	
H8A	0.3542	−0.1143	0.5415	0.025*	
C9	0.3237 (3)	−0.00778 (11)	0.56601 (15)	0.0179 (4)	
C10	0.2217 (3)	0.00785 (12)	0.47635 (16)	0.0217 (4)	
H10A	0.1787	0.0537	0.4692	0.026*	
C11	0.1837 (3)	−0.03853 (11)	0.40215 (16)	0.0197 (4)	
H11A	0.2259	−0.0845	0.4089	0.024*	
C12	0.0821 (3)	−0.02164 (11)	0.31320 (16)	0.0197 (4)	
C13	0.0397 (3)	−0.06890 (11)	0.23773 (17)	0.0210 (4)	
H13A	0.0709	−0.1166	0.2387	0.025*	
C14	−0.0574 (3)	−0.03622 (13)	0.15811 (18)	0.0261 (5)	
H14A	−0.0965	−0.0603	0.1010	0.031*	
C15	−0.0870 (3)	0.03424 (13)	0.17442 (18)	0.0258 (5)	
H15A	−0.1489	0.0635	0.1301	0.031*	
C16	0.3192 (3)	0.11898 (12)	0.60670 (18)	0.0254 (5)	
H16A	0.3170	0.1256	0.5360	0.038*	
H16B	0.4009	0.1507	0.6383	0.038*	
H16C	0.2091	0.1288	0.6300	0.038*	
Br1	1.28408 (3)	0.278131 (13)	0.464999 (17)	0.02491 (7)	
S2	0.66919 (6)	0.23502 (2)	0.13641 (3)	0.01276 (9)	
O1	0.51250 (19)	0.23380 (8)	0.18823 (12)	0.0197 (3)	
O2	0.6865 (2)	0.29873 (8)	0.07625 (11)	0.0202 (3)	
O3	0.7004 (2)	0.16993 (8)	0.08184 (11)	0.0211 (3)	
C17	0.8027 (3)	0.25412 (11)	0.32873 (15)	0.0177 (4)	
H17A	0.6913	0.2560	0.3474	0.021*	
C18	0.9364 (3)	0.26436 (12)	0.39958 (15)	0.0203 (4)	
H18A	0.9153	0.2731	0.4656	0.024*	
C19	1.1011 (3)	0.26130 (11)	0.36972 (15)	0.0174 (4)	
C20	1.1376 (3)	0.24670 (11)	0.27242 (16)	0.0173 (4)	
H20A	1.2492	0.2436	0.2544	0.021*	
C21	1.0033 (3)	0.23684 (10)	0.20250 (15)	0.0161 (4)	
H21A	1.0251	0.2274	0.1367	0.019*	
C22	0.8362 (2)	0.24106 (10)	0.23020 (14)	0.0141 (3)	

### Atomic displacement parameters (Å^2^)

**Table:**

	*U*^11^	*U*^22^	*U*^33^	*U*^12^	*U*^13^	*U*^23^
S1	0.0243 (3)	0.0173 (2)	0.0262 (3)	0.00428 (19)	0.0006 (2)	0.00228 (19)
N1	0.0176 (8)	0.0160 (8)	0.0201 (8)	−0.0014 (6)	0.0019 (6)	−0.0001 (6)
C1	0.0151 (9)	0.0204 (9)	0.0182 (9)	−0.0018 (7)	0.0033 (7)	−0.0004 (7)
C2	0.0207 (10)	0.0209 (10)	0.0243 (10)	−0.0012 (8)	0.0026 (8)	−0.0038 (8)
C3	0.0239 (11)	0.0281 (11)	0.0220 (10)	−0.0047 (9)	0.0020 (8)	−0.0083 (8)
C4	0.0229 (11)	0.0358 (12)	0.0183 (10)	−0.0039 (9)	0.0000 (8)	0.0013 (9)
C5	0.0206 (10)	0.0230 (10)	0.0226 (10)	−0.0011 (8)	0.0026 (8)	0.0053 (8)
C6	0.0170 (9)	0.0211 (10)	0.0177 (9)	−0.0025 (7)	0.0035 (7)	0.0001 (7)
C7	0.0218 (10)	0.0154 (9)	0.0222 (10)	−0.0007 (7)	0.0026 (8)	0.0016 (7)
C8	0.0235 (10)	0.0160 (9)	0.0217 (10)	−0.0026 (8)	0.0013 (8)	−0.0030 (7)
C9	0.0176 (9)	0.0173 (9)	0.0189 (9)	−0.0023 (7)	0.0027 (7)	0.0001 (7)
C10	0.0237 (10)	0.0192 (9)	0.0221 (10)	0.0007 (8)	0.0007 (8)	0.0001 (8)
C11	0.0191 (10)	0.0179 (9)	0.0222 (10)	0.0000 (7)	0.0021 (8)	0.0020 (7)
C12	0.0178 (9)	0.0180 (9)	0.0232 (10)	0.0002 (7)	0.0002 (8)	0.0040 (8)
C13	0.0195 (10)	0.0158 (9)	0.0279 (11)	−0.0001 (7)	0.0024 (8)	0.0010 (8)
C14	0.0260 (11)	0.0250 (11)	0.0266 (11)	−0.0017 (9)	−0.0039 (9)	0.0001 (9)
C15	0.0219 (10)	0.0273 (11)	0.0274 (11)	0.0046 (9)	−0.0056 (8)	0.0065 (9)
C16	0.0293 (11)	0.0152 (9)	0.0310 (11)	−0.0001 (8)	−0.0046 (9)	−0.0004 (8)
Br1	0.02009 (11)	0.03105 (12)	0.02239 (11)	−0.00586 (8)	−0.00934 (8)	0.00605 (8)
S2	0.0121 (2)	0.0129 (2)	0.01296 (19)	−0.00040 (15)	−0.00194 (15)	0.00143 (15)
O1	0.0130 (7)	0.0228 (7)	0.0230 (7)	−0.0007 (5)	−0.0006 (5)	0.0033 (6)
O2	0.0221 (7)	0.0189 (7)	0.0193 (7)	−0.0012 (6)	−0.0031 (6)	0.0072 (6)
O3	0.0228 (7)	0.0187 (7)	0.0211 (7)	0.0018 (6)	−0.0048 (6)	−0.0048 (6)
C17	0.0147 (9)	0.0231 (10)	0.0156 (9)	−0.0020 (7)	0.0022 (7)	0.0008 (7)
C18	0.0206 (10)	0.0269 (10)	0.0132 (8)	−0.0032 (8)	−0.0004 (7)	0.0011 (7)
C19	0.0141 (9)	0.0196 (9)	0.0178 (9)	−0.0032 (7)	−0.0058 (7)	0.0041 (7)
C20	0.0118 (8)	0.0178 (9)	0.0221 (9)	0.0002 (7)	−0.0001 (7)	0.0030 (7)
C21	0.0153 (9)	0.0168 (9)	0.0164 (8)	−0.0001 (7)	0.0016 (7)	0.0003 (7)
C22	0.0138 (8)	0.0123 (8)	0.0160 (8)	−0.0005 (6)	−0.0014 (7)	0.0008 (6)

### Geometric parameters (Å, °)

**Table:**

S1—C15	1.705 (2)	C12—C13	1.378 (3)
S1—C12	1.731 (2)	C13—C14	1.423 (3)
N1—C9	1.350 (3)	C13—H13A	0.9300
N1—C1	1.402 (3)	C14—C15	1.367 (3)
N1—C16	1.481 (3)	C14—H14A	0.9300
C1—C6	1.407 (3)	C15—H15A	0.9300
C1—C2	1.410 (3)	C16—H16A	0.9600
C2—C3	1.370 (3)	C16—H16B	0.9600
C2—H2A	0.9300	C16—H16C	0.9600
C3—C4	1.409 (4)	Br1—C19	1.9050 (19)
C3—H3A	0.9300	S2—O1	1.4565 (16)
C4—C5	1.376 (3)	S2—O3	1.4572 (15)
C4—H4A	0.9300	S2—O2	1.4585 (15)
C5—C6	1.412 (3)	S2—C22	1.778 (2)
C5—H5A	0.9300	C17—C22	1.391 (3)
C6—C7	1.417 (3)	C17—C18	1.396 (3)
C7—C8	1.357 (3)	C17—H17A	0.9300
C7—H7A	0.9300	C18—C19	1.385 (3)
C8—C9	1.421 (3)	C18—H18A	0.9300
C8—H8A	0.9300	C19—C20	1.387 (3)
C9—C10	1.444 (3)	C20—C21	1.391 (3)
C10—C11	1.348 (3)	C20—H20A	0.9300
C10—H10A	0.9300	C21—C22	1.395 (3)
C11—C12	1.440 (3)	C21—H21A	0.9300
C11—H11A	0.9300		
			
C15—S1—C12	91.95 (11)	C12—C13—C14	112.1 (2)
C9—N1—C1	122.04 (18)	C12—C13—H13A	124.0
C9—N1—C16	119.69 (18)	C14—C13—H13A	124.0
C1—N1—C16	118.27 (18)	C15—C14—C13	112.8 (2)
N1—C1—C6	118.71 (18)	C15—C14—H14A	123.6
N1—C1—C2	121.65 (19)	C13—C14—H14A	123.6
C6—C1—C2	119.6 (2)	C14—C15—S1	112.02 (17)
C3—C2—C1	119.4 (2)	C14—C15—H15A	124.0
C3—C2—H2A	120.3	S1—C15—H15A	124.0
C1—C2—H2A	120.3	N1—C16—H16A	109.5
C2—C3—C4	121.6 (2)	N1—C16—H16B	109.5
C2—C3—H3A	119.2	H16A—C16—H16B	109.5
C4—C3—H3A	119.2	N1—C16—H16C	109.5
C5—C4—C3	119.7 (2)	H16A—C16—H16C	109.5
C5—C4—H4A	120.2	H16B—C16—H16C	109.5
C3—C4—H4A	120.2	O1—S2—O3	113.71 (9)
C4—C5—C6	119.9 (2)	O1—S2—O2	112.89 (9)
C4—C5—H5A	120.0	O3—S2—O2	112.74 (9)
C6—C5—H5A	120.0	O1—S2—C22	106.05 (9)
C1—C6—C5	119.8 (2)	O3—S2—C22	105.82 (9)
C1—C6—C7	119.02 (19)	O2—S2—C22	104.69 (9)
C5—C6—C7	121.1 (2)	C22—C17—C18	119.99 (19)
C8—C7—C6	120.5 (2)	C22—C17—H17A	120.0
C8—C7—H7A	119.8	C18—C17—H17A	120.0
C6—C7—H7A	119.8	C19—C18—C17	118.81 (19)
C7—C8—C9	120.48 (19)	C19—C18—H18A	120.6
C7—C8—H8A	119.8	C17—C18—H18A	120.6
C9—C8—H8A	119.8	C18—C19—C20	122.23 (18)
N1—C9—C8	119.19 (19)	C18—C19—Br1	119.13 (16)
N1—C9—C10	120.16 (19)	C20—C19—Br1	118.64 (15)
C8—C9—C10	120.65 (19)	C19—C20—C21	118.37 (18)
C11—C10—C9	125.4 (2)	C19—C20—H20A	120.8
C11—C10—H10A	117.3	C21—C20—H20A	120.8
C9—C10—H10A	117.3	C20—C21—C22	120.55 (18)
C10—C11—C12	124.4 (2)	C20—C21—H21A	119.7
C10—C11—H11A	117.8	C22—C21—H21A	119.7
C12—C11—H11A	117.8	C17—C22—C21	120.02 (18)
C13—C12—C11	124.9 (2)	C17—C22—S2	121.09 (15)
C13—C12—S1	111.13 (16)	C21—C22—S2	118.77 (15)
C11—C12—S1	123.91 (17)		
			
C9—N1—C1—C6	2.6 (3)	C9—C10—C11—C12	−179.8 (2)
C16—N1—C1—C6	−177.04 (19)	C10—C11—C12—C13	−178.7 (2)
C9—N1—C1—C2	−176.27 (19)	C10—C11—C12—S1	3.4 (3)
C16—N1—C1—C2	4.1 (3)	C15—S1—C12—C13	0.22 (18)
N1—C1—C2—C3	178.8 (2)	C15—S1—C12—C11	178.3 (2)
C6—C1—C2—C3	0.0 (3)	C11—C12—C13—C14	−178.2 (2)
C1—C2—C3—C4	−0.1 (3)	S1—C12—C13—C14	−0.1 (2)
C2—C3—C4—C5	−0.1 (4)	C12—C13—C14—C15	−0.2 (3)
C3—C4—C5—C6	0.5 (3)	C13—C14—C15—S1	0.3 (3)
N1—C1—C6—C5	−178.50 (18)	C12—S1—C15—C14	−0.3 (2)
C2—C1—C6—C5	0.4 (3)	C22—C17—C18—C19	0.1 (3)
N1—C1—C6—C7	0.4 (3)	C17—C18—C19—C20	−1.6 (3)
C2—C1—C6—C7	179.31 (19)	C17—C18—C19—Br1	178.06 (16)
C4—C5—C6—C1	−0.6 (3)	C18—C19—C20—C21	1.8 (3)
C4—C5—C6—C7	−179.5 (2)	Br1—C19—C20—C21	−177.86 (15)
C1—C6—C7—C8	−2.0 (3)	C19—C20—C21—C22	−0.5 (3)
C5—C6—C7—C8	176.9 (2)	C18—C17—C22—C21	1.2 (3)
C6—C7—C8—C9	0.7 (3)	C18—C17—C22—S2	−174.80 (16)
C1—N1—C9—C8	−3.9 (3)	C20—C21—C22—C17	−1.0 (3)
C16—N1—C9—C8	175.7 (2)	C20—C21—C22—S2	175.11 (15)
C1—N1—C9—C10	176.08 (19)	O1—S2—C22—C17	−9.57 (19)
C16—N1—C9—C10	−4.3 (3)	O3—S2—C22—C17	−130.66 (17)
C7—C8—C9—N1	2.2 (3)	O2—S2—C22—C17	110.03 (17)
C7—C8—C9—C10	−177.8 (2)	O1—S2—C22—C21	174.37 (15)
N1—C9—C10—C11	173.8 (2)	O3—S2—C22—C21	53.28 (18)
C8—C9—C10—C11	−6.2 (3)	O2—S2—C22—C21	−66.04 (17)

### Hydrogen-bond geometry (Å, °)

**Table:**

Cg4 is the centroid of the C17–C22 benzene ring.

**Table:**

*D*—H···*A*	*D*—H	H···*A*	*D*···*A*	*D*—H···*A*
C3—H3A···O3^i^	0.93	2.51	3.409 (3)	162
C7—H7A···O1^ii^	0.93	2.47	3.241 (3)	140
C8—H8A···O2^iii^	0.93	2.29	3.218 (3)	175
C10—H10A···S1	0.93	2.77	3.185 (2)	108
C11—H11A···O2^iii^	0.93	2.31	3.238 (3)	176
C15—H15A···O3^iv^	0.93	2.40	3.265 (3)	154
C16—H16B···O1^v^	0.96	2.43	3.321 (3)	155
C17—H17A···O1	0.93	2.54	2.915 (3)	105
C20—H20A···O1^vi^	0.93	2.32	3.242 (3)	172
C13—H13A···Cg4^iii^	0.93	2.57	3.434 (2)	155

Symmetry codes: (i) *x*, *y*, *z*+1; (ii) −*x*+1, −*y*, −*z*+1; (iii) −*x*+1, *y*−1/2, −*z*+1/2;  (iv) *x*−1, *y*, *z*; (v) *x*, −*y*+1/2, *z*+1/2; (vi) *x*+1, *y*, *z*.
